# A Regional Scale Approach to Assessing Current and Potential Future Exposure to Tidal Inundation in Different Types of Estuaries

**DOI:** 10.1038/s41598-018-25410-y

**Published:** 2018-05-04

**Authors:** David J. Hanslow, Bradley D. Morris, Edwina Foulsham, Michael A. Kinsela

**Affiliations:** Science Division, Office of Environment and Heritage, NSW Government, 59 Goulburn Street, Sydney, New South Wales 2000 Australia

## Abstract

Broad scale assessments of impacts associated with sea level rise have mainly been undertaken using ocean water level data from tide gauges located in harbours and ports assuming that these can be applied directly in mapping inundation throughout estuaries. On many coasts, however, exposure to sea level rise comes about through inundation adjacent to rivers and estuaries, in many instances far from the ocean. In this study, we examine the potential impacts of sea level rise within the diverse estuaries of South East Australia. We use an extensive and long-term water level data set, which show that water levels within the different types of estuaries vary from ocean water levels. We map potential inundation scenarios for each estuary using an approach which improves on the commonly used bath tub method by allowing for variation in tidal processes both between and along estuaries. We identify considerable exposure to future sea level rise, and variable suitability of the bath tub method within different estuaries. Exposure is particularly high around tidal lake systems, where reduced tidal ranges have allowed development to occur in relative proximity to present sea level, and around larger coastal rivers, which feature extensive low-lying plains exposed to potential inundation.

## Introduction

The potential impact of sea level rise on coastal and island communities is one of the most concerning elements of climate change. By 2100 global mean sea level (GMSL) is projected to increase (*likely* range, indicative of the central 66% percentile range of projections) under a business as usual greenhouse gas scenario (Representative Concentration Pathway RCP 8.5) by between 0.52 and 0.98 m, or 0.26 to 0.55 m with significantly reduced emissions (RCP 2.6) relative to 1986–2005^1^. Beyond the likely range, a recent review by the US National Oceanic and Atmospheric Administration (NOAA)^2^ identifies evidence in support of a physically plausible GMSL rise in the range of 2.0 to 2.7 m by 2100, with recent work on Antarctic ice-sheet instability indicating that such outcomes may be more likely than previously thought. Sea level rise is not uniformly distributed and most coastlines around the world are projected to experience sea level change within about 20% of the global average^1^. For South East Australia, mean model predictions suggest sea level rise of around 0–10% above the global average^1^, giving a projected *likely* range of 0.54 to 1.06 m for RCP 8.5 or 0.24 to 0.61 m for RCP 2.6^3^.

Beyond 2100, the Intergovernmental Panel on Climate Change (IPCC)^4^ conclude that it is virtually certain that global mean sea level rise will continue for many centuries due to thermal expansion of the oceans and (probably underestimated) ongoing contributions from the loss of mass from ice sheets. Recent projections^5^ indicate under RCP 8.5, a *very likely* range (indicative of the central 90% percentile range of projections) of 1.0 to 3.7 m by 2200, which under strong emissions mitigation (RCP 2.6) is reduced to 0.3 to 2.4 m. The IPCC^4^ predict warming greater than a threshold above 1 °C but less than about 4 °C would lead to the near-complete loss of the Greenland ice sheet. This would result in a global mean sea level rise of up to 7 m over a millennium or more. They also suggest the possibility of abrupt and irreversible ice loss from a potential instability of marine based sectors of the Antarctic ice sheet in response to climate forcing. Satellite observations on the West Antarctic ice sheet indicate a general acceleration in ice loss from west Antarctica, including grounding line retreat of significant glaciers^6,7^. Numerical modelling suggests that ice-sheet collapse has already begun to occur in the Thwaites Glacier Basin^8^. Recent modelling suggests that collapse is inevitable under mid to high range global warming scenarios^6^, and that this area could contribute significantly to sea level rise in the decades and centuries to come^6^. The recent review by NOAA^2^ provides a lower limit of 0.39 m and an upper limit estimate of 9.7 m sea level rise by 2200. Beyond 2200 Antarctica has the potential to contribute more sea level rise if greenhouse gas emissions continue unabated^9^.

Globally, hundreds of millions of people are vulnerable to rising sea levels^10–12^. Potential impacts include: higher and more frequent storm surges and oceanic inundation events, with eventual permanent inundation of low lying areas^13,14^; landward recession of sandy shorelines^15^; salt water intrusion^16^ and landward advance of tidal limits within estuaries^17^; failure of stormwater infrastructure and sewerage systems^18^; and, modification of river/estuary entrance dynamics as well as catchment flood behaviour^18,19^. Exposure of current development to inundation and erosion is likely to gradually increase over time, with rates of sea level rise expected to accelerate within the present century^4^. At the same time, the level of protection provided by existing seawalls and coastal defences will potentially decrease, due to the increasing threat from storm surges and inundation at higher projected water levels.

On open coasts, there is usually some level of natural defence against ocean inundation, typically through the presence of headlands and coastal beach-dune barrier systems. This has, to date, generally limited significant impacts to only the more extreme events. While these settings are threatened by sea level rise and potential future changes to wave climate, the intermittent and variable nature of extreme events makes it difficult to detect and attribute impacts that are enhanced by climate change^6,20^. Within estuaries, however, many settlements are located in very low-lying areas and thus are highly vulnerable to sea level rise. Here the creeping effects of sea level rise are becoming apparent through the growing frequency of ‘nuisance inundation’ or ‘sunny day flooding’^14,21,22^.

A variety of approaches have been taken to assess the exposure of communities to the potential impacts of inundation caused by sea level rise. These vary from broad-scale assessments based on elevation above sea level^23–26^_,_ in some instances adjusted to allow for variations in ocean tides and storm surge^27–29^, or using interpolation between measured gauge data^30–32^, to detailed local studies using hydrodynamic modelling^23,33^. Generally, the scale of the assessment determines the level of detail in the methodology^24,25^, with the reliability of the results dependent on the approach taken relative to the complexity of processes operating in the particular setting.

In Australia thus far, broad scale exposure assessments^28,29^ have generally only considered variations in open coast tide and surge levels, and have ignored variation in tides within estuaries, even though estuarine water levels are known to vary from ocean water levels^34–36^. These assessments have highlighted a significant future problem with greatest exposure concentrated in South Eastern Australia^28,29^. Here the open coast is characterised by headlands and dunes, which limit exposure to inundation during extreme events^37,38^, but is backed by extensive estuarine waterways that are typically surrounded by low-lying coastal plains. To evaluate and refine previous assessments of potential impacts from sea level rise in South Eastern Australia, we describe an improved method to quantify current and potential future exposure of property and infrastructure, which addresses the variation in tidal water levels within different types of estuaries. Our study quantifies the limitations of the much relied upon ‘bath tub’ method, and provides data to inform strategic land use planning and to assess the need for, and prioritisation of, adaptation planning and action.

There are 184 significant estuaries along the New South Wales (NSW) coast^35,39,40^. Modification of tides within estuaries can include tidal lag, tidal distortion, elevation of half tide levels or tidal pumping, and amplification of fortnightly tides^36^. The tidal range in estuaries is known to be affected by factors including inertia related to acceleration and deceleration effects, amplification associated with the decrease of the width and depth (convergence), damping due to bottom friction, and partial reflection at abrupt changes in bathymetry^41–45^. These processes result in fundamentally different patterns of tidal behaviour in different estuaries^36,46^. Some estuaries experience tidal amplification while others are characterised by tidal attenuation^36,42^, or a combination of both. In drowned river valley estuaries, estuary geometry generally deepens and widens in a seaward direction. This promotes tidal amplification and increased tide range and height for considerable distances inland^36^. Within tidal rivers, river entrance shoals contribute to initial attenuation of the tide, followed by mild amplification before complete damping at fluvial gravel and sand bars around the head of the estuary^36^. Tidal lakes are characterised by severe attenuation of the tidal range due to frictional effects in the entrance channel. Tide ranges in these systems may be as little as 10% of the offshore tide range^36^ whilst tidal pumping can significantly amplify the magnitude of the fortnightly tide^43,47^. Smaller lake systems are usually characterised by intermittent entrance opening and closing and are known as Intermittently Closed and Open Lakes and Lagoons (ICOLLs). While open, they operate like tidal lakes. While closed, they gradually fill, with water levels influenced by inflows and evaporation. In these systems, maximum water levels are generally controlled by beach berm height^48–50^.

To assess the exposure of existing development (i.e. property and infrastructure) to inundation from potential sea level rise we use tidal plane levels from an extensive set of tide gauges published by Manly Hydraulics Laboratory (MHL)^51^. We adopt the High High Water Solstice Springs (HHWSS) tidal plane for water surface mapping in all estuary types, except coastal lakes with mostly closed entrances, where berm height is used to approximate inundation potential. We apply a method which allows for variation in water levels both between and within individual estuaries. Tidal plane surfaces are overlain on high resolution digital elevation models to map inundation extent. The impacts associated with sea level rise scenarios of 0.5 m, 1.0 m and 1.5 m are assessed.

## Results

**Figure 1:**
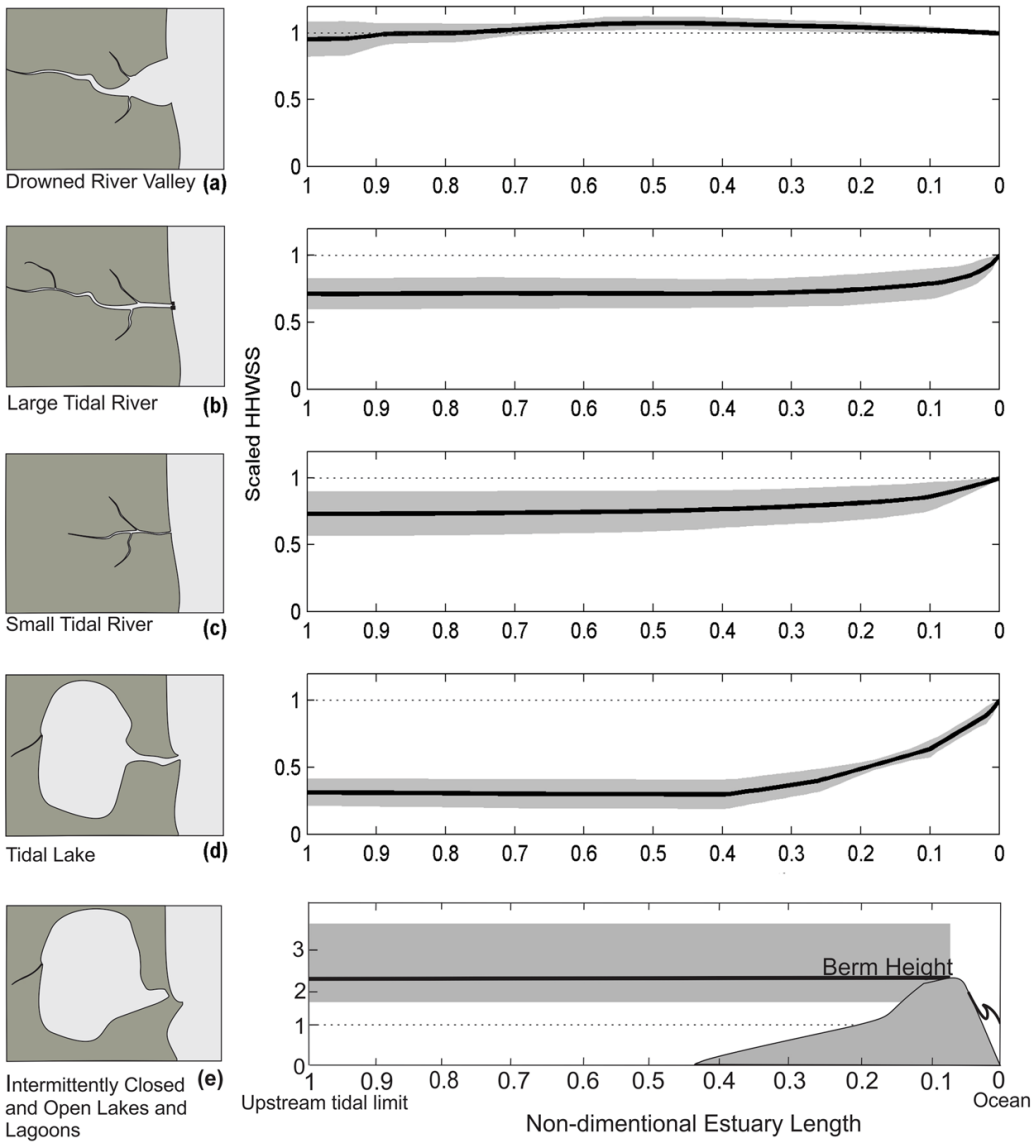
Average dimensionless tidal planes for five of the six estuary types used in this study. The sixth type were ocean embayment’s, where no tidal modification was assumed. The grey envelopes show the bounds of tide gauge data for all gauged estuaries of each estuary type. The dotted lines show the equivalent tidal plane using a bath tub approach. The non-dimensional estuary length is calculated by scaling the actual estuary length for each estuary.

The variation in the HHWSS tidal plane both within and between different estuary types is shown in Fig. 1. Here we plot the non-dimensional tidal plane height (scaled against the ocean tidal plane level adjacent to the entrance) with non-dimensional estuary length. We also show the equivalent tidal plane adopting a bath tub assumption. The data show tidal water levels within drowned river valleys are subject to mild amplification compared with the ocean, while small and large rivers show varying degrees of attenuation. Greatest tidal attenuation occurs in tidal lakes where water level is significantly lower than the ocean. Within ICOLL’s, the berm height controls maximum water levels when the inlet is closed, and is always elevated above the ocean tide level because of the role of wave runup in berm development^48^. The finding that water levels within different estuary types vary from those seen in the ocean is not new^41,43,45,46^ but does not seem to have been widely appreciated or applied in many broad scale sea level rise risk assessments. However, one example of the application of gauge data from some estuaries within a broad-scale assessment is the recent assessment in the in the US^32^. This risk assessment used mapping undertaken by NOAA^30,31^ which is developed using water surfaces interpolated between tide gauges including some within estuaries.

**Figure 2:**
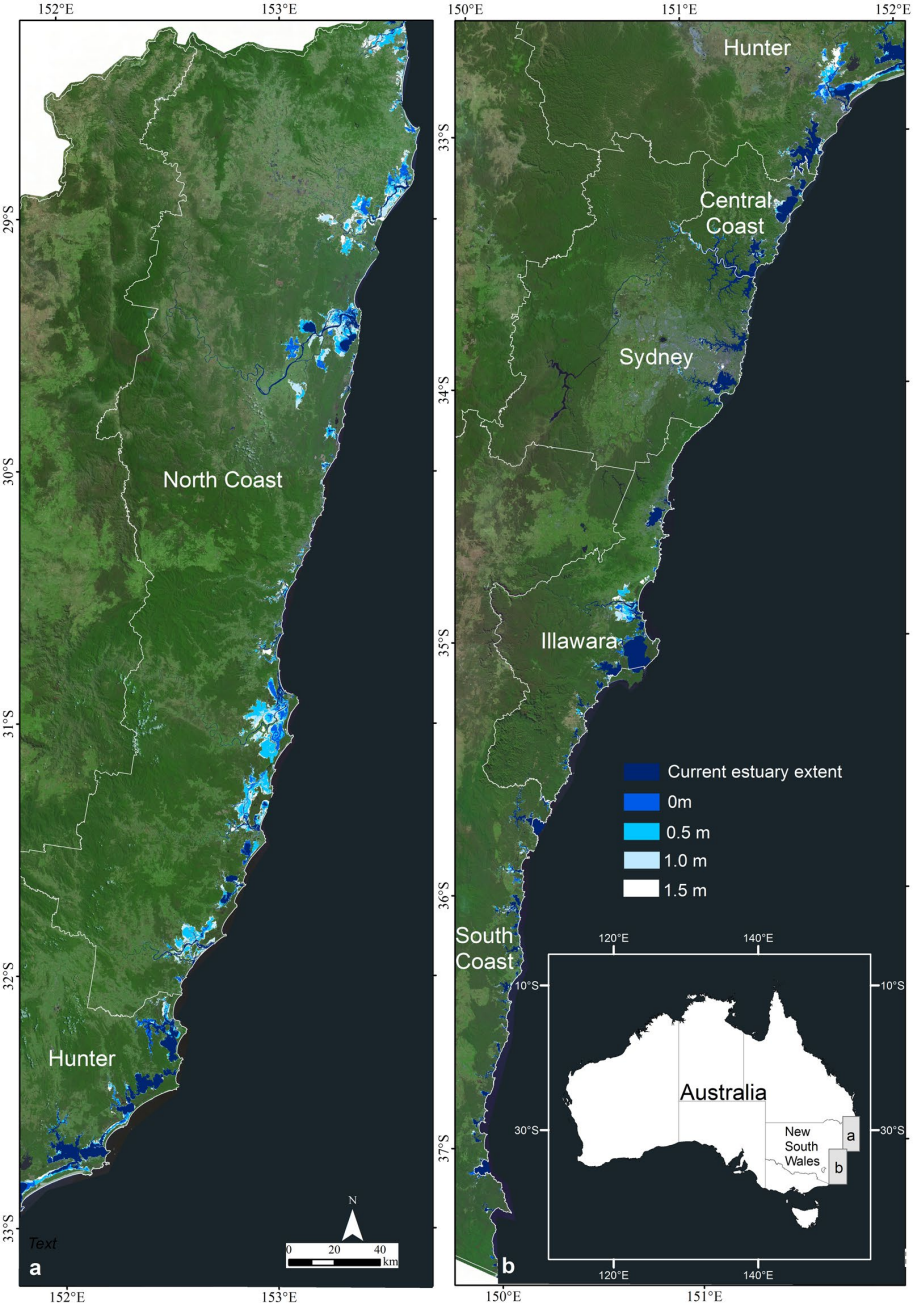
Inundation mapping for the 0 m (current HHWSS or berm level), 0.5 m, 1.0 m and 1.5 m sea level rise scenarios together with the current mapped extent for all 184 estuaries in New South Wales and the regional breakup used in the exposure assessment. This figure was created using ARCGIS v10.4 http://desktop.arcgis.com/en/arcmap/.

**Figure 3:**
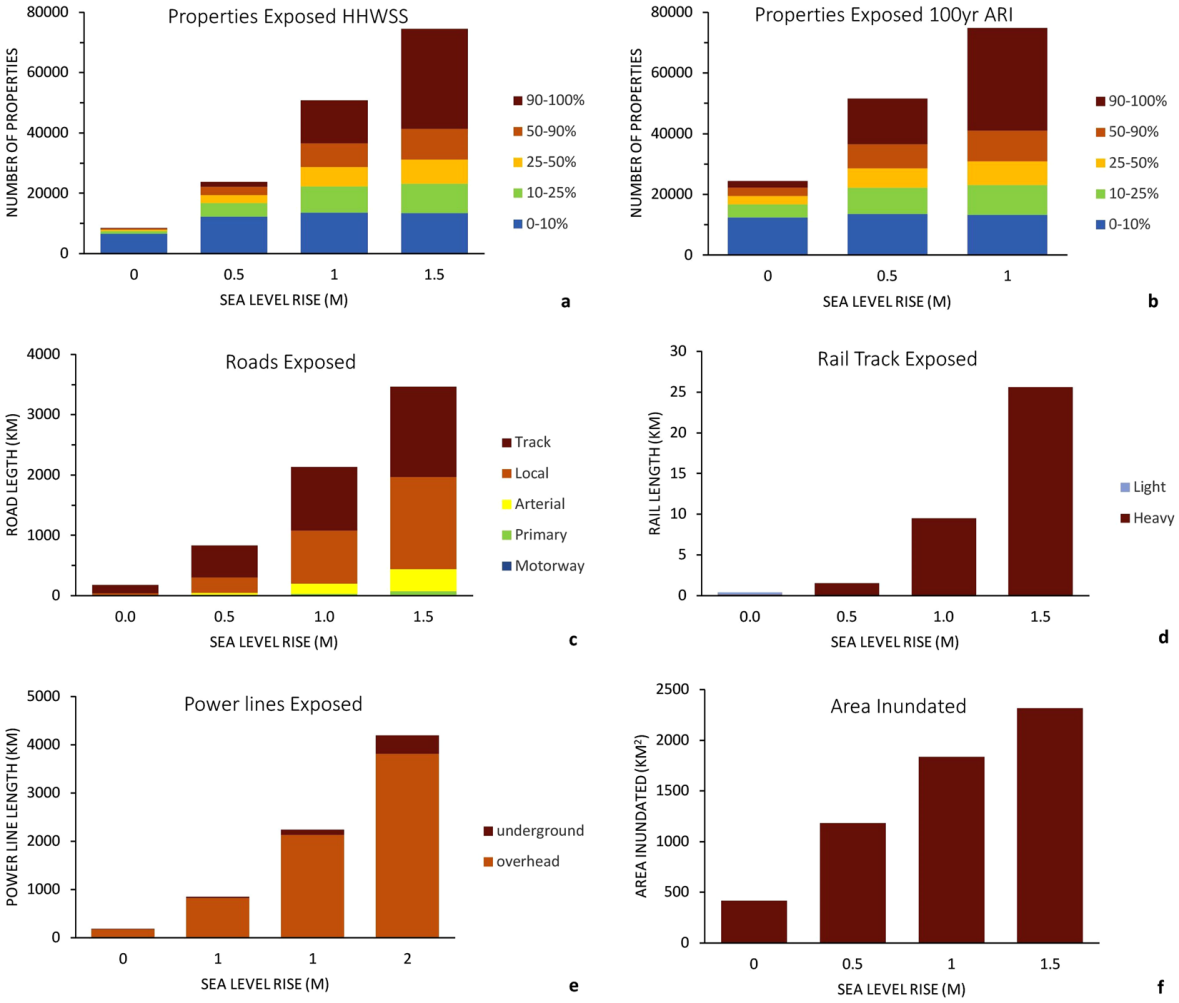
Overall exposure results including: (**a**) Total numbers of properties exposed to inundation (HHWSS or berm level) under 0 m, 0.5 m, 1.0 m and 1.5 m sea level rise scenarios including the proportion of property land area inundated (0–10%, 10–25%, 25–50%, 50–90% and 90–100%); (**b**) total numbers of properties exposed to inundation at the 100 yr. ARI level under 0 m, 0.5 m, and 1.0 m sea level rise scenarios including the proportion of property land area inundated (0–10%, 10–25%, 25–50%, 50–90% and 90–100%); (**c**) length of road exposed to inundation (HHWSS or berm level) under 0 m, 0.5 m, 1.0 m and 1.5 m sea level rise scenarios including road type (track, local, arterial, primary or motorway); (**d**) length of rail track exposed to inundation (HHWSS or berm level) under 0 m, 0.5 m, 1.0 m and 1.5 m sea level rise scenarios including track type (light, heavy); (**e**) length of power line exposed to inundation (HHWSS or berm level) under 0 m, 0.5 m, 1.0 m and 1.5 m sea level rise scenarios including line type (underground, overhead); (**f**) overall area inundated (HHWSS or berm level) under 0 m, 0.5 m, 1.0 m and 1.5 m sea level rise scenarios.

Mapping of the current estuary inundation extent (0 m) at the HHWSS or berm level in mostly closed ICOLLs and for the 0.5, 1.0 and 1.5 m sea level rise scenarios in all 184 estuaries in NSW is shown in Fig. 2 with the measured inundation areas provided in Supplementary Table s1. The current mapped inundation extent in most estuaries is similar but slightly larger than the existing mapped estuary area, as would be expected as the existing mapped estuary extents are referenced to mean high water. The main exceptions to this occur in the larger river systems on the North Coast (Richmond River, Clarence River and Macleay River) and in the Hunter region (Hunter River) which are fringed by extensive low-lying areas. Overall, some 413, 1181, 1836, 2315 km^2^ of land beyond the current mapped extent of these estuaries is subject to inundation for the four water level scenarios respectively. Most inundation occurs in the northern part of the NSW coast with large areas of inundation adjacent to the larger river systems. At the current HHWSS level, inundation throughout all NSW estuaries impacts some 8500 properties (Fig. 3a). Exposure increases to some 23700 properties if sea level rises by 0.5 m, 50700 if sea level rises by 1 m and 74400 if sea level rises by 1.5 m. Currently the extent of inundation of most properties is only minor (i.e. <10% land area inundated), although as sea levels rise, the proportion of properties subject to major or complete inundation increases. Of the current properties exposed to inundation, only 600 are subject to greater than 50% land-area inundation, and 200 to greater than 90% land-area inundation. This increases to 4300 and 1600 respectively for 0.5 m of sea level rise, 22100 and 14200 for 1 m of sea level rise, and 43300 and 33100 for 1.5 m of sea level rise.

Allowing for storm surge and other non-tidal contributors to ocean water levels (≈100 yr. Annual Return Interval (ARI)), we find some 24300 properties are currently exposed (Fig. 3b). This increases to 51600 properties if sea level rises by 0.5 m and 74700 properties if sea level rises by 1 m (Fig. 3). Of the current properties exposed to inundation, only 5000 are subject to greater than 50% land-area inundation. This increases to 23000 for 0.5 m of sea level rise and 43900 for 1 m of sea level rise. Only 2200 properties are currently subject to greater than 90% land-area inundation. This increases to 15100 for 0.5 m of sea level rise and 33800 for 1 m of sea level rise.

Exposure of roads, railways, power lines and airfields sea level rise is quantified in Fig. 3c–f. Exposure of road infrastructure is greatest with up to 3458 km of roads potentially subject to inundation with 1.5 m of sea level rise. Local roads and tracks make up most of this exposure although some arterial and primary roads are also impacted under the higher sea level rise scenarios. Similar quantities of power infrastructure are potentially exposed, as electricity lines are usually paired with roadways. However, as most power infrastructure is above ground it is unlikely to be significantly impacted.

**Figure 4:**
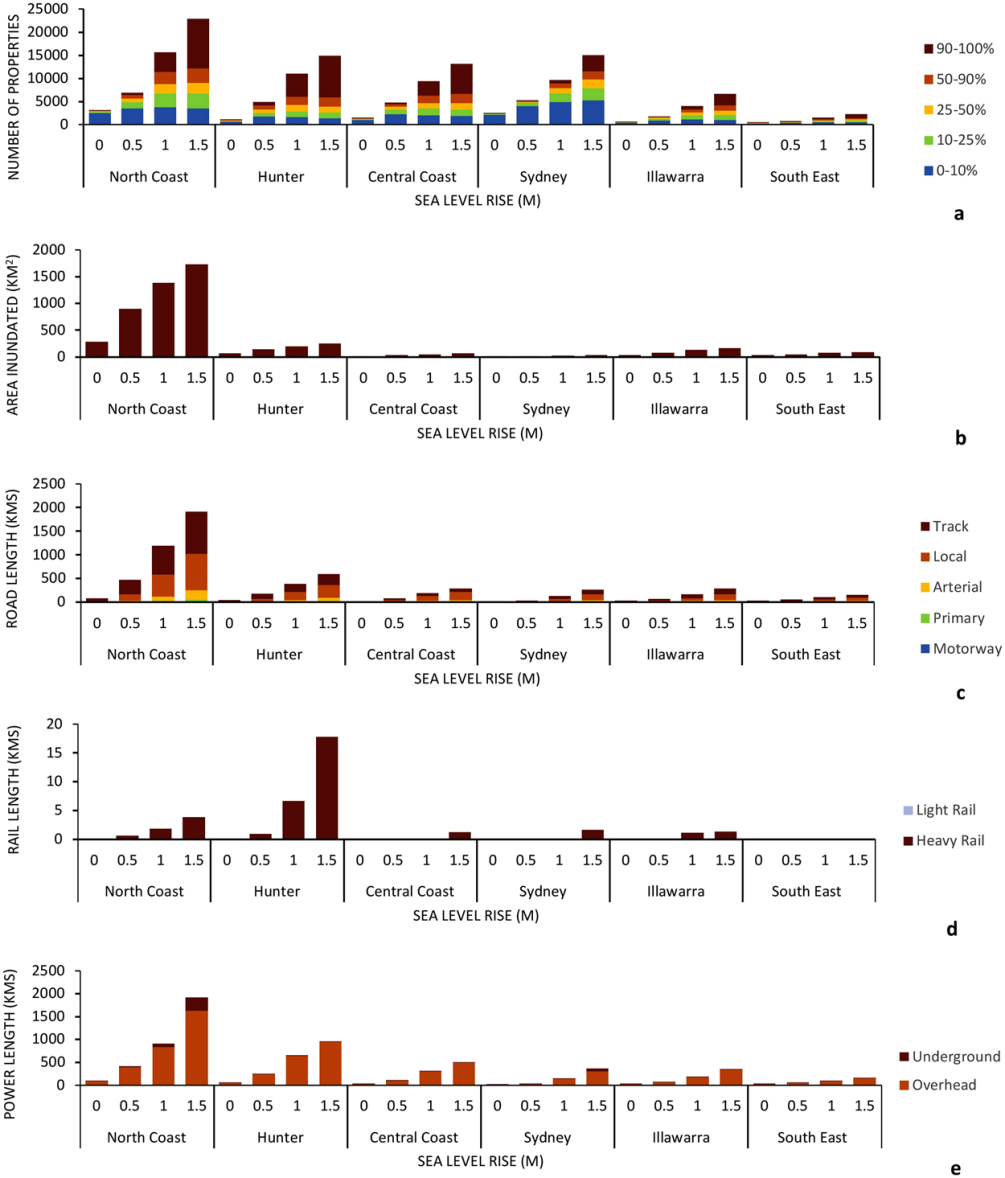
Regional spread in exposure results including. (**a**) Total numbers of properties exposed to inundation (HHWSS or berm level) under 0 m, 0.5 m, 1.0 m and 1.5 m sea level rise scenarios including the proportion of property land area inundated (0–10%, 10–25%, 25–50%, 50–90% and 90–100%); (**b**) overall area inundated (HHWSS or berm level) under 0 m, 0.5 m, 1.0 m and 1.5 m sea level rise scenarios; (**c**) length of road exposed to inundation (HHWSS or berm level) under 0 m, 0.5 m, 1.0 m and 1.5 m sea level rise scenarios including road type (track, local, arterial, primary or motorway); (**d**) length of rail track exposed to inundation (HHWSS or berm level) under 0 m, 0.5 m, 1.0 m and 1.5 m sea level rise scenarios including track type (light, heavy); (**e**) length of power line exposed to inundation (HHWSS or berm level) under 0 m, 0.5 m, 1.0 m and 1.5 m sea level rise scenarios including line type (underground, overhead).

The regional spread of exposure of properties, the land area inundated, as well as the length of roads, rail and power infrastructure is shown in Fig. 4. Most exposure occurs on the North Coast, although significant numbers properties are also exposed in the Hunter, Central Coast and Sydney regions. The least exposed region is the south coast, which is generally less developed and features smaller coastal lakes and lagoons surrounded by rugged hinterland.

**Figure 5:**
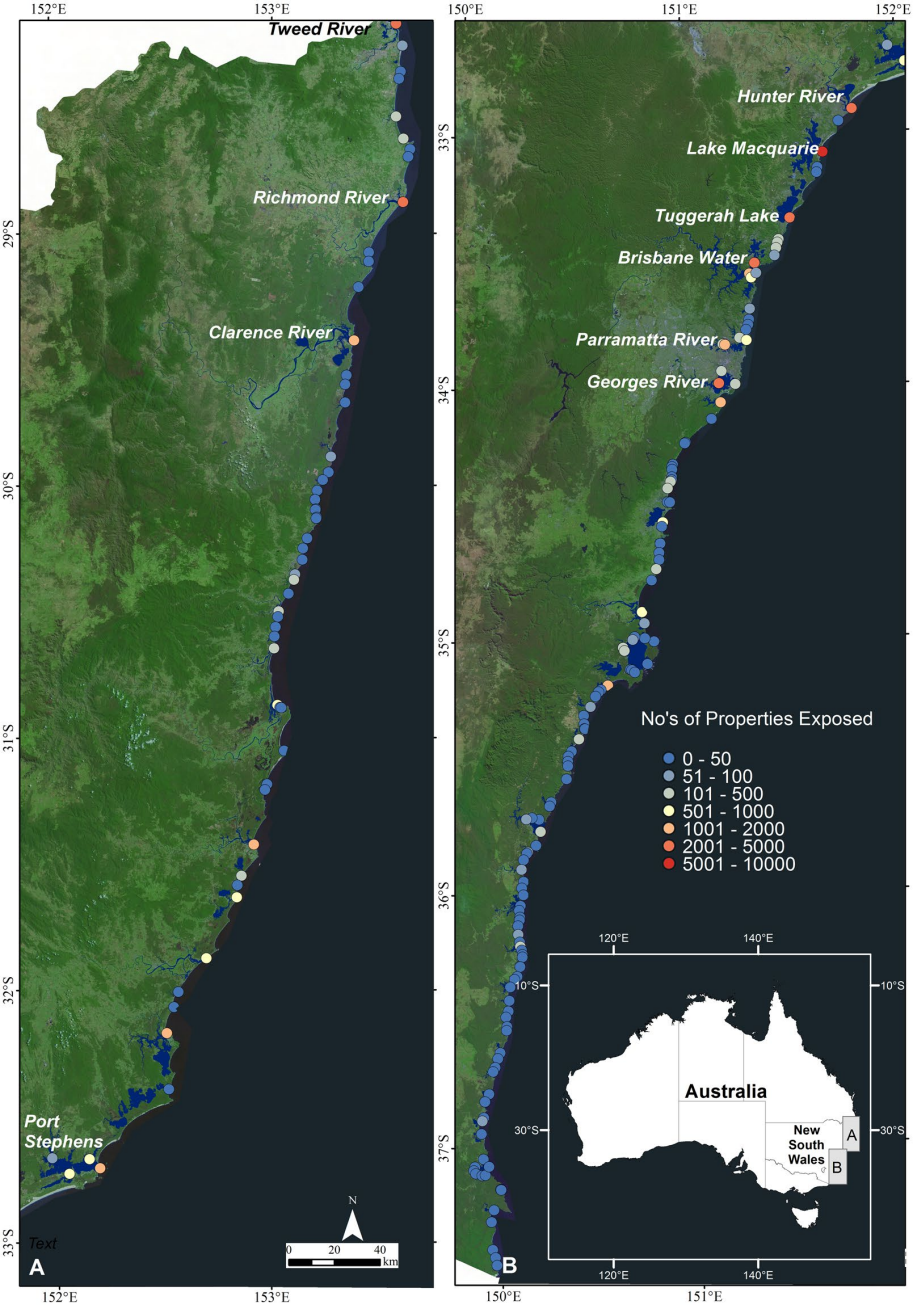
Geographical spread of exposure for the 1 m sea level rise scenario and locations of the 10 most exposed estuaries. This figure was created using ARCGIS v10.4 http://desktop.arcgis.com/en/arcmap/.

**Figure 6:**
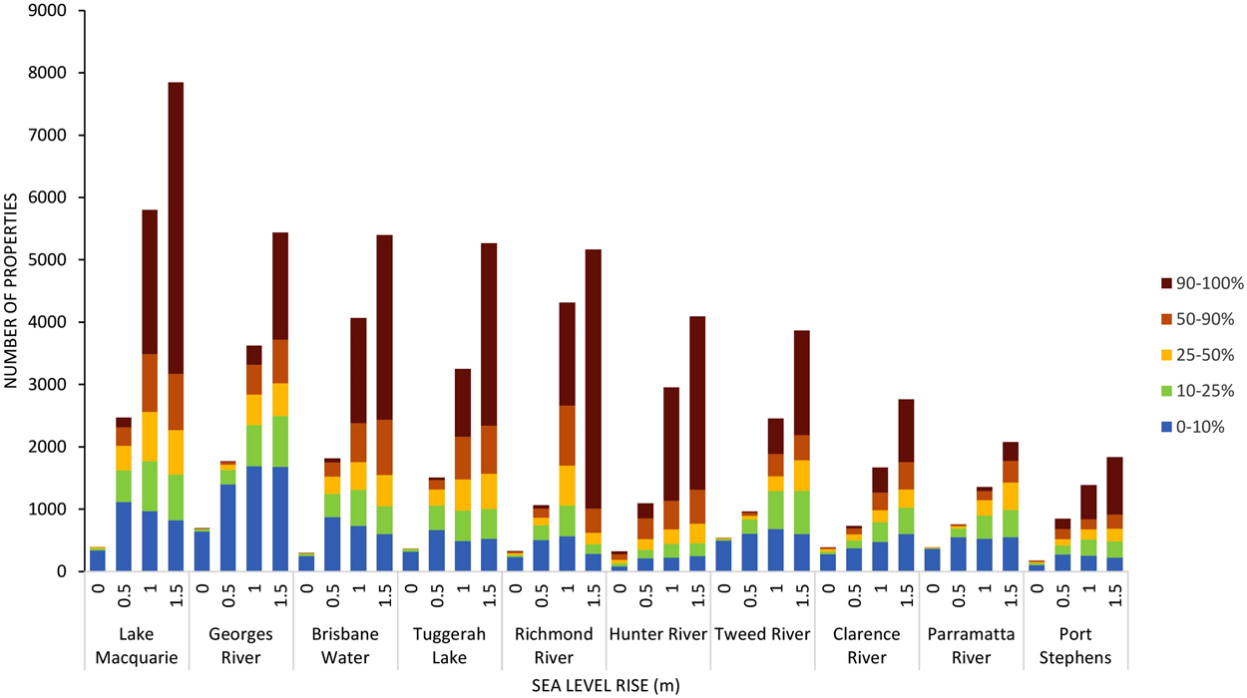
Total numbers of properties exposed to inundation (HHWSS) for the 10 most exposed estuaries under 0 m, 0.5 m, 1.0 m and 1.5 m sea level rise scenarios including the proportion of property land area inundated (0–10%, 10–25%, 25–50%, 50–90% and 90–100%).

The geographical spread of exposure for the 1 m sea level rise scenario is shown in Fig. 5, as the total number of exposed properties surrounding each estuary. The 10 most exposed estuaries include Lake Macquarie, Georges River, Brisbane Water, Tuggerah Lake, Richmond River, Hunter River, Tweed River, Clarence River, Parramatta River, and Port Stephens (Fig. 6). These estuaries are coastal lakes, larger rivers and drowned river valleys, and account for 61% of the overall exposure throughout NSW.

## Discussion

Our results show a considerable number of properties and infrastructure along the NSW coast are exposed to the potential impacts from inundation from sea level rise and broadly confirm earlier findings from the Australian National Coastal Risk Assessment^28,29^ which was undertaken to examine the extent of risks posed by climate change within the Australian coastal zone. However, our results also capture complexity in the distribution of exposure, emerging not only from the elevation and concentration of development, but also from estuary morphology. Our results show the current extent of inundation of most properties is only minor (i.e. <10% land area inundated), although as sea levels rise, the proportion of properties subject to major or complete inundation increases. This comes about through the progressive inundation of, and eventual complete inundation of, lower lying properties at the forefront of exposure. We show that greatest exposure occurs around tidal lakes and adjacent to the larger and more heavily populated coastal river systems (Fig. 2). Within tidal lake systems, reduced tidal range in combination with extensive coastal plains, has promoted development in relative proximity to sea level. Much of this development has been shown to be only marginally above current high tide levels and thus is highly vulnerable to sea level rise. The extensive nature of the coastal rivers and the location of several major towns on surrounding river plains also contributes to exposure to sea level rise within the tidal river systems.

**Figure 7:**
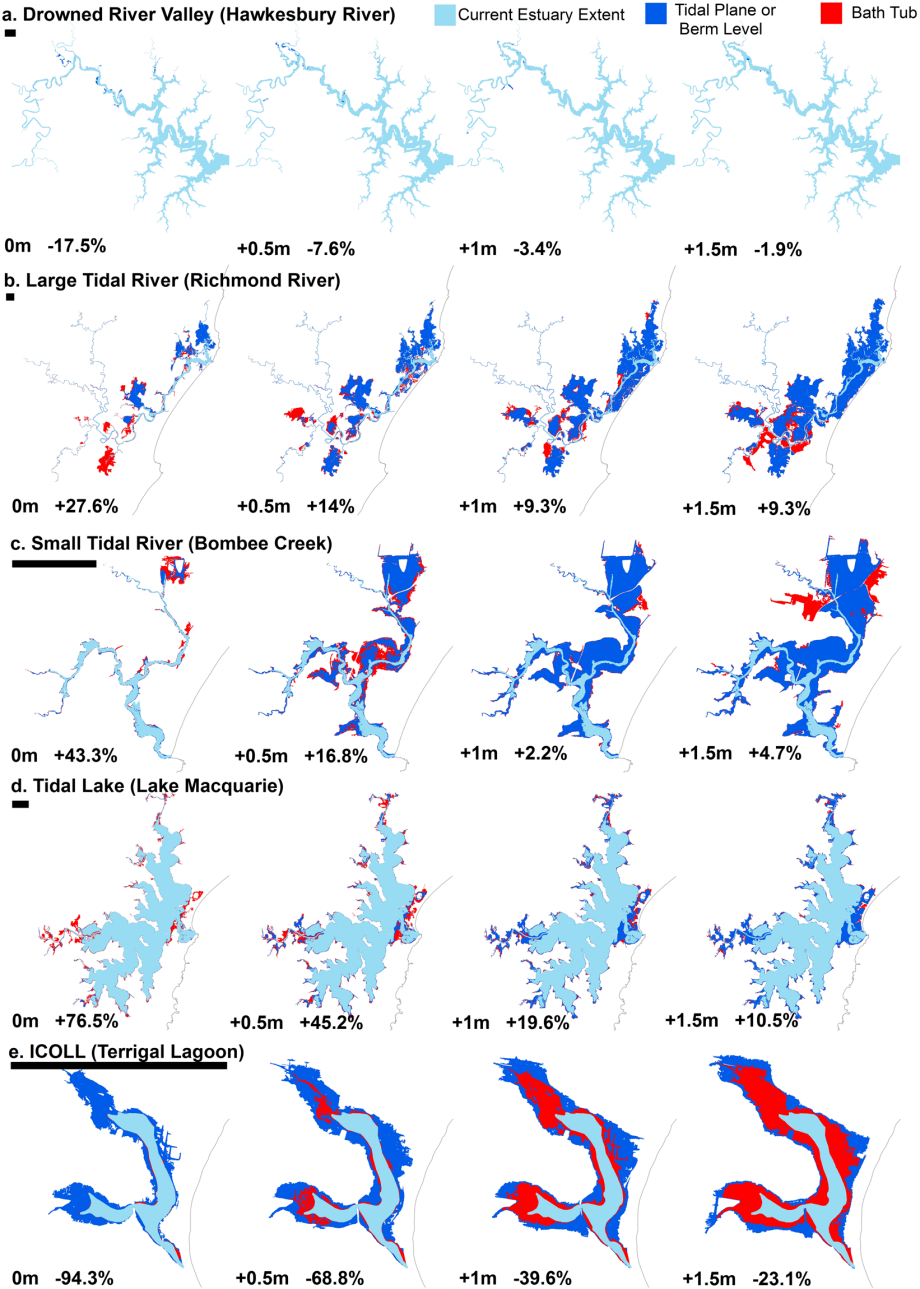
Comparison between the predicted inundation extent using the tidal plane surface method or berm height (blue) and the bath tub approach (red) for different estuary types (**a**) drowned river valley -Hawkesbury River, (**b**) large tidal river -Richmond River, (**c**) small tidal river -Boambee Creek, (**d**) tidal lake -Lake Macquarie, (**e**) ICOLL -Terrigal Lagoon). Mapping is presented for the 0 m (current HHWSS or berm level in Terrigal Lagoon), 0.5 m, 1.0 m and 1.5 m sea level rise scenarios together with the current estuary mapped extent (light blue). The percent difference in the mapped inundation extent based on the bath tub model, relative to the inundation extent based on the tidal plane surface method or berm height, is indicated for each scenario. The comparative scale bar for each estuary is 2 km. This figure was created using ARCGIS v10.4 http://desktop.arcgis.com/en/arcmap/.

The tidal plane data used in this study suggests previous bath tub type assessments would, however, have potentially underestimated exposure in the mid reaches of drowned river valleys and within ICOLL’s and overestimated exposure in tidal lake and river type estuaries (Fig. 1). To confirm this, we compared the predicted area of inundation using our tidal plane mapping method with a bath tub approach, within a typical example of each estuary type for each inundation scenario. The results of this comparison are shown in Fig. 7. For the drowned diver valley example (Hawkesbury River) a bath tub approach underestimates the extent of current HHWSS inundation by some 18% compared with the tidal plane method. Within large (Richmond River) and small tidal rivers (Boambee Creek) the bath tub method over estimates the current extent of inundation by some 28% and 43% respectively. In Lake Macquarie (Tidal Lake) the bathtub over estimates the extent of current inundation by some 77%. Within Terrigal Lagoon (ICOLL) the bath tub approach using current HHWSS results in 94% less inundation than the use of the berm height. Under the sea level rise scenarios, the differences remain consistent but diminishes with larger sea level rise scenarios. This has broader implications than just within South East Australian estuaries which, while diverse, only form a sub-set of global estuarine diversity^41,42,44^. The significance of this finding will likely vary with coastal setting and depend on estuary type, surrounding geomorphology and level of development as well as tide range. Globally there is significant variability in estuarine types and thus it would be expected similar variability will occur in other settings highlighting the need to consider variations in water levels both between and within different estuary types when considering potential vulnerability to tidal inundation.

Lake Macquarie was identified as the most exposed estuary in South East Australia which is consistent with the findings of the Australian National Coastal Risk Assessment^28,29^ that identified this local government area as the most exposed in Australia. That assessment was undertaken as a first pass assessment of exposure to sea level rise using a simple bath tub or bucket fill approach. While it is difficult to directly compare the current exposure assessment with the national risk assessment, the findings seem similar, which on preliminary assessment is somewhat surprising given the difference in methodology used. The national risk assessment found between 5,100 and 6,800 buildings in the local government area of Lake Macquarie may be affected by sea-level rise and storm tide inundation by 2100 as compared with 5580 (at least 50% of lot inundation) in the current study with 1 m of sea level rise and assuming 0.5 m of storm surge. Here we assume the 50% of lot measure is similar to the centroid of lot measure used in the national risk assessment. While the actual water levels used in the national risk assessment are not disclosed, the application of a 1.1 m sea level rise allowance in combination with a 100 yr. storm surge and the assumption that water levels inside the lake match those in the ocean would be expected to have significantly over estimated exposure in this system compared with other types of estuaries, particularly drowned river valleys. The results of the comparison between methods seen in Fig. 7c does however indicate a reduction in the difference in the predicted area of inundation (to 10.5%) with higher sea level rise scenarios. This pattern was also seen in other estuary types and is attributed to a reduction in the proportion of land adjacent each estuary with increasing elevation.

Comparatively, our results show the potential future growth of exposure to inundation within estuaries to be a far greater problem in NSW than that of exposure to open coast erosion, which while nevertheless significant^52^, is an order of magnitude less than the results presented here. Overall this exposure assessment finds esturine tidal inundation to be a major coastal management issue, highlighting the need for coastal and floodplain management and planning to manage risk to current development and to avoid the unnecessary growth of risk into the future.

## Methods

We assess exposure of current development to inundation under current conditions and with sea level rise scenarios of 0.5, 1.0 and 1.5 m. Offshore tidal levels along the coast of NSW are derived from the Pacific Ocean 1/12° data set^53,54^. We use this model because several of the available tide gauge records along the coast of South East Australia are located within river entrances and are not fully representative of ocean tide conditions^55^. For tidal inundation, the High High Water Solstice Springs (HHWSS) tidal plane is adopted for all estuary types except normally closed ICOLLs, as this tidal plane is available for all tide gauges in South Eastern Australia^51^. For each tide gauge, MHL^51^ calculated tidal constituents and tidal planes from 1990–2010 using the Foreman tidal analysis package^56,57^. HHWSS is given by Z_0_ + M_2 + _S_2_ + 1.4*(K_1_ + O_1_), where Z_0_ is the mean sea level adjusted to Australian Height Datum (AHD), M_2_ is the principle semi-diurnal lunar tidal constituent, S_2_ is the principle semi-diurnal solar constituent, K_1_ is the luni-solar diurnal constituent and O_1_ is the principal lunar diurnal constituent. The HHWSS tidal plane was originally defined as the level beyond which tides seldom reach^58^. It is consistent with predicted levels for higher (king) tides but is slightly lower than highest astronomical tide (HAT).

The 0.5, 1.0 and 1.5 m sea level rise scenarios capture a range of possible magnitudes for sea level rise within the present century and beyond^1–5^. A 0.5 m sea level rise scenario also approximates the magnitude of a 100 yr ARI surge allowance on the NSW coast^59,60^ and is therefore used to estimate exposure to storm surge and other non-tidal contributors to ocean water levels in all estuary types except ICOLL’s where berm height is used to map the inundation extent. Supplementary Figure s1 plots a comparison between the 100 yr. ARI levels using published data^60^ and the HHWSS tidal plane at both offshore (30 m Water depth) and bay and harbour tide gauge locations along the NSW coast. On average the 100 yr. ARI water level is around 0.4–0.5 m above the respective HHWSS tidal plane and is fairly consistent along the NSW coast. This allowance however, excludes effects of wave setup and wave runup which may be significant at some sites.

Offshore tide levels are combined with published tidal plane levels from 180 tide gauges in 56 estuaries^51^ for current estuarine water levels in gauged estuaries. This data is also used to categorise NSW estuary planes and identify characteristic tidal plane types for application to non-gauged estuaries. Average non-dimensional tidal planes are calculated for each estuary type by using available data scaled using the entrance tidal elevation and the measured estuary length^61^. Estuary types used include embayment’s (where ocean tide levels are applied), drowned river valleys, small and large rivers, tidal lakes and intermittently open and closed lakes and lagoons following^35–37^ (see Fig. 1). In tidal lakes, to address tidal pumping and amplification of the fortnightly tide^62^ we add an additional 0.2 m to the tidal plane level based on exceedance statistics from MHL^63^. Within ICOLLs with entrances which are usually closed we use berm height measurements where available or a formulation for berm height^37,48^. For the 100 yr. ARI calculation we use berm height for all ICOLLs.

Inundation extents are mapped by overlying tidal plane surfaces fitted utilising a minimum curvature spline technique on 1 m digital elevation model data (DEM) derived from LiDAR data^64,65^. The DEM’s have a horizontal accuracy of 0.8 m and vertical accuracy of 0.3 m (95% CI). The resultant inundation extents are compared with the existing mapped extent of each estuary to evaluate the mapped inundation extent. Additionally, we compare the predicted inundation extent using the tidal plane surface or berm height method with a bath tub approach for a typical example of each estuary type. In this comparison the bath tub extent is mapped using the entrance HHWSS. The percent difference in the mapped extent relative to the extent of the tidal plane surface method or berm height is calculated for each scenario.

The geocoded urban and rural address system (GURAS)^66^ was used to identify properties that were predicted to be exposed to inundation under each scenario using a similar approach to that adopted in the recent 2^nd^ pass erosion assessment^52^. The GURAS database stores address point data within cadastral lots. The database distinguishes between primary addresses (e.g. houses, strata blocks) and secondary addresses (e.g. individual apartments or units). Only primary addresses were considered in this assessment. The GURAS database also contains coded text identifiers which were used to select and discard a variety of public-space and utilities address types prior to the calculation of exposure statistics. A procedure was developed to restrict the assessment only to address types of interest, which include houses, unit or apartment blocks (but not individual units within), and commercial and industrial premises. The procedure removed secondary addresses, address points falling within Crown Lands and National Parks, and other unwanted address types (e.g. beaches, reserves, car parks, wharfs, utilities etc.). The land-area proportion of each property allotment (i.e. <10%, 10–25%, 25–50%; 50–90% and 90–100%) that was predicted to be exposed to inundation was also calculated, and appended to each address point to provide an assessment of the degree of inundation of each property. The exposure of transport (roads, rail) and electricity infrastructure to inundation was also quantified using an overlay method, which identified road, railway, and power line-geometry data that fell within the inundation hazard extents. For each scenario, total lengths of potentially exposed infrastructure were calculated. The transport infrastructure the assessment was limited to road, railway and pathway lengths that were classified as ‘on ground’, and thus excluded any raised (bridges, overpasses) or buried (tunnels) infrastructure.

## Limitations

Our approach, while making a significant advancement by allowing for variation in water levels between and along individual estuaries using long-term tide gauge records, remains a broad scale assessment and does not replace the need to undertake high resolution flood or inundation studies for individual estuaries. The HHWSS tidal plane is on average 0.15 m below HAT^67^ and thus does not represent the full extent of tidal inundation. Additionally, this tidal plane does not include non-tidal processes including storm surge, although the 0.5 m sea level offset is representative of a first order allowance for 100 yr. ARI non-tidal water level variations (excluding effects of wave setup, runup and coincident rainfall related flooding) as seen in Supplementary Figure s1. Within gauged estuaries the planes are limited by the distribution of gauges and the accuracy of the formulations used to calculate the plane. In ungauged estuaries, we adopt planes from gauged estuaries of the same estuary type, thus assuming average conditions by type (Fig. 1). Conveyance of waters within some estuaries is limited by flood gates which are not mapped and thus not included in this assessment. To address conveyance issues, we restrict inundation between areas where connectivity is limited, by excluding areas which are separated by more than 5 m. This may however underestimate inundation where conveyance is allowed via storm water systems. We do not consider the impact of sea level rise combined with fluvial flooding (or its potential to increase with a warming climate) as this is highly variable and requires detailed modelling. There is considerable existing exposure to fluvial flood related processes in NSW estuaries thus the current study significantly underestimates total current exposure. We expect the overall exposure to tidal and fluvial flooding to increase with sea level rise and possibly increased intensity of extreme rainfall.

The three sea level rise scenarios considered are selected to be representative of a range of future sea levels relevant to structure design as well as land use planning. They are not tied to particular planning horizons, but are consistent with Intermediate-Low, Intermediate, Intermediate-High scenarios by 2100 released by NOAA^5^. Importantly the adopted scenarios should not be considered an upper bound for either sea level rise by 2100 or potential long-term sea level rise which may need to be considered for future development. Sea level rises are added to the existing tidal planes and thus assume no change in tidal range or form. Recent studies^46,68,69^ have shown that shelf, coastal and estuarine tides are potentially subject to change with sea level rise, although these changes are likely to be small compared with the magnitude of sea level rise. Additionally, we assume that the geomorphology of estuaries and their tidal inlets remains unchanged with future sea level, except within ICOLLs where an increase in berm height is applied. Vertical accretion of wetlands may offset some impacts of sea level rise particularly at lower rates^70–72^, however this is not expected to significantly impact the exposure of property quantified in this study.

Exposure is quantified using data from the GURAS database. Therefore, our method only examines the exposure of property allotments and not the actual elevation of assets, and the exposure assessment findings may overestimate actual exposure of assets. The exposure assessment is limited to broad scale quantification of the inundation of property and infrastructure. Many impacts on the environment and ecosystem services are also likely with sea level rise have not been considered in this assessment.

### Data Availability

The tidal plane data used in this study is available from Manly Hydraulics Laboratory^51^ http://new.mhl.nsw.gov.au/services/publications/oehreport. The LiDAR data was sourced from Land and Property Information and is available from http://elevation.fsdf.org.au/. A mapping viewer containing the inundation layers produced as part of the study is currently under development.

## Electronic supplementary material

Supplementary Information
