# Supplementary material for: A Regional Scale Approach to Assessing Current and Potential Future Exposure to Tidal Inundation in Different Types of Estuaries
Source: Sci Rep. 2018 May 4;8:7065. doi: 10.1038/s41598-018-25410-y (PMC5935728; doi:10.1038/s41598-018-25410-y)
Supplement: Supplementary file 1 — Supplementary Information [file 41598_2018_25410_MOESM1_ESM.docx]

**A Regional Scale Approach to Assessing Current and Potential Future Exposure to Tidal Inundation in Different Types of Estuaries.**

David. J. Hanslow^1^, Bradley. D. Morris^1^, Edwina Foulsham^1^ and Michael. A. Kinsela^1^

^1^Science Division, Office of Environment and Heritage, NSW Government, 59 Goulburn Street, Sydney, New South Wales 2000, Australia, david.hanslow@environment.nsw.gov.au

**Supplementary Figure S1** Along coast variation in the HHWSS tidal plane^48^ and extreme (100yr ARI) water levels^57^ at offshore (labelled in blue) and bay or harbour gauging locations (labelled in black).

**Supplementary Table s1**

| Estuary | Existing Mapped Estuary Area (km²) | 0m Mapped  Area (km^2^) | +0.5m Mapped Area (km^2^) | +1.0m Mapped Area (km^2^) | +1.5m  Mapped Area (km^2^) |
| --- | --- | --- | --- | --- | --- |
| 1 - Tweed River | 22.73 | 28.07 | 65.36 | 92.05 | 120.84 |
| 2 - Cudgen Creek | 2.15 | 4.22 | 8.66 | 14.02 | 16.58 |
| 3 - Cudgera Creek | 0.49 | 0.81 | 3.63 | 5.80 | 7.18 |
| 4 - Mooball Creek | 0.54 | 0.82 | 1.39 | 6.61 | 13.11 |
| 5 - Brunswick River | 3.61 | 4.20 | 8.58 | 13.58 | 18.20 |
| 6 - Belongil Creek | 0.27 | 6.33 | 8.55 | 10.64 | 13.80 |
| 7 - Tallow Creek | 0.12 | 0.19 | 0.51 | 0.65 | 0.80 |
| 8 - Broken Head Creek | 0.05 | 0.05 | 0.12 | 0.18 | 0.22 |
| 9 - Richmond River | 38.41 | 85.42 | 172.50 | 259.84 | 339.59 |
| 10 - Salty Lagoon | 0.16 | 0.48 | 0.93 | 1.20 | 1.43 |
| 11 - Evans River | 2.66 | 3.29 | 5.36 | 8.46 | 10.25 |
| 12 - Jerusalem Creek | 0.32 | 0.48 | 0.58 | 0.73 | 0.86 |
| 13 - Clarence River | 132.34 | 192.95 | 281.90 | 383.89 | 423.28 |
| 14 - Lake Arragan | 0.97 | 1.01 | 1.66 | 2.15 | 2.70 |
| 15 - Cakora Lagoon | 0.36 | 0.81 | 1.18 | 1.55 | 2.12 |
| 16 - Sandon River | 2.63 | 3.07 | 3.81 | 5.18 | 6.92 |
| 17 - Wooli River | 3.76 | 5.67 | 14.32 | 19.32 | 22.25 |
| 18 - Station Creek | 0.26 | 0.67 | 0.89 | 1.07 | 1.22 |
| 19 - Corindi River | 1.91 | 3.40 | 6.11 | 8.08 | 9.71 |
| 20 - Pipe Clay Creek | 0.01 | 0.01 | 0.02 | 0.02 | 0.03 |
| 21 - Arrawarra Creek | 0.13 | 0.27 | 0.53 | 1.20 | 1.67 |
| 22 - Darkum Creek | 0.06 | 0.09 | 0.19 | 0.28 | 0.42 |
| 23 - Woolgoolga Lake | 0.16 | 0.16 | 0.17 | 0.30 | 0.43 |
| 24 - Flat Top Point Creek | 0.02 | 0.11 | 0.20 | 0.25 | 0.29 |
| 25 - Hearns Lake | 0.15 | 0.17 | 0.25 | 0.35 | 0.50 |
| 26 - Moonee Creek | 0.65 | 0.81 | 1.00 | 1.64 | 2.06 |
| 27 - Pine Brush Creek | 0.02 | 0.05 | 0.06 | 0.09 | 0.12 |
| 28 - Coffs Creek | 0.46 | 0.54 | 0.62 | 0.80 | 1.03 |
| 29 - Boambee Creek | 1.00 | 1.18 | 2.86 | 4.35 | 5.31 |
| 30 - Bonville Creek | 1.66 | 2.01 | 2.71 | 5.09 | 7.02 |
| 31 - Bundageree Creek | 0.01 | 0.34 | 0.47 | 0.55 | 0.63 |
| 32 - Bellinger River | 8.17 | 9.29 | 13.70 | 22.76 | 29.92 |
| 33 - Dalhousie Creek | 0.08 | 0.14 | 0.16 | 0.19 | 0.23 |
| 34 - Oyster Creek | 0.14 | 0.25 | 0.29 | 0.36 | 0.44 |
| 35 - Deep Creek | 1.72 | 1.88 | 2.54 | 3.73 | 4.54 |
| 36 - Nambucca River | 12.65 | 14.79 | 22.89 | 28.81 | 41.88 |
| 37 - Macleay River | 31.65 | 92.76 | 225.93 | 297.37 | 334.92 |
| 38 - South West Rocks Creek | 0.94 | 1.10 | 1.27 | 1.36 | 1.42 |
| 39 - Saltwater Creek Frederickton | 0.28 | 0.84 | 1.05 | 1.22 | 1.53 |
| 40 - Korogoro Creek | 0.28 | 0.35 | 0.43 | 0.94 | 1.52 |
| 41 - Killick Creek | 0.29 | 0.36 | 0.42 | 0.88 | 1.33 |
| 42 - Goolawah Lagoon | 0.13 | 0.13 | 0.59 | 1.14 | 1.28 |
| 43 - Hastings River | 29.97 | 49.00 | 133.22 | 185.08 | 218.02 |
| 44 - Cathie Creek | 13.75 | 24.55 | 33.46 | 36.19 | 38.23 |
| 45 - Duchess Gully | 0.02 | 0.07 | 0.09 | 0.10 | 0.19 |
| 46 - Camden Haven River | 32.16 | 32.93 | 41.82 | 53.37 | 59.14 |
| 47 - Manning River | 34.74 | 42.73 | 105.46 | 154.31 | 184.04 |
| 48 - Khappinghat Creek | 1.19 | 4.18 | 5.13 | 6.19 | 7.30 |
| 49 - Black Head Lagoon | 0.01 | 0.05 | 0.07 | 0.12 | 0.17 |
| 50 - Wallis Lake | 98.71 | 124.56 | 145.57 | 161.65 | 172.00 |
| 51 - Smiths Lake | 10.01 | 11.20 | 11.52 | 11.86 | 12.23 |
| 52 - Myall River | 115.20 | 130.17 | 150.07 | 163.51 | 177.20 |
| 53 - Karuah River | 17.88 | 19.56 | 26.60 | 28.74 | 29.93 |
| 54 - Tilligerry Creek | 20.46 | 24.94 | 42.32 | 50.25 | 58.20 |
| 55 - Port Stephens | 134.38 | 137.27 | 145.80 | 151.98 | 156.07 |
| 56 - Hunter River | 55.26 | 108.79 | 149.24 | 182.55 | 221.04 |
| 57 - Glenrock Lagoon | 0.05 | 0.07 | 0.08 | 0.08 | 0.09 |
| 58 - Lake Macquarie | 116.16 | 117.51 | 123.79 | 134.85 | 142.68 |
| 59 - Middle Camp Creek | 0.01 | 0.07 | 0.11 | 0.14 | 0.17 |
| 60 - Moonee Beach Creek | 0.00 | 0.01 | 0.01 | 0.01 | 0.01 |
| 61 - Tuggerah Lake | 80.93 | 82.06 | 86.81 | 95.96 | 104.59 |
| 62 - Wamberal Lagoon | 0.52 | 0.77 | 0.88 | 1.01 | 1.14 |
| 63 - Terrigal Lagoon | 0.28 | 0.62 | 0.82 | 0.96 | 1.08 |
| 64 - Avoca Lake | 0.67 | 1.05 | 1.18 | 1.27 | 1.34 |
| 65 - Cockrone Lake | 0.33 | 0.48 | 0.56 | 0.62 | 0.67 |
| 66 - Brisbane Water | 30.60 | 31.07 | 33.24 | 37.19 | 39.88 |
| 67 - Hawkesbury River | 119.65 | 126.05 | 136.25 | 142.95 | 147.52 |
| 68 - Pittwater | 18.46 | 18.94 | 19.26 | 19.71 | 20.18 |
| 69 - BrokenBay | 0.01 | 0.15 | 0.21 | 0.28 | 0.34 |
| 70 - Narrabeen Lagoon | 2.33 | 2.34 | 2.46 | 2.93 | 3.50 |
| 71 - Dee Why Lagoon | 0.32 | 0.39 | 0.47 | 0.58 | 0.67 |
| 72 - Curl Lagoon | 0.07 | 0.11 | 0.18 | 0.23 | 0.29 |
| 73 - Manly Lagoon | 0.10 | 0.10 | 0.12 | 0.26 | 0.65 |
| 74 - Middle Harbour Creek | 6.21 | 6.61 | 6.73 | 6.91 | 7.11 |
| 75 - Lane Cove River | 3.08 | 3.23 | 3.35 | 3.53 | 3.68 |
| 76 - Parramatta River | 14.41 | 15.11 | 15.61 | 17.26 | 19.24 |
| 77 - PortJackson | 29.07 | 29.55 | 29.74 | 30.14 | 31.50 |
| 78 - Cooks River | 1.27 | 1.35 | 1.67 | 2.82 | 5.73 |
| 79 - Georges River | 28.63 | 30.22 | 32.54 | 36.68 | 41.34 |
| 80 - BotanyBay | 40.63 | 41.13 | 41.55 | 42.12 | 43.17 |
| 81 - Port Hacking | 12.01 | 12.65 | 12.88 | 13.49 | 13.82 |
| 82 - Wattamolla Creek | 0.03 | 0.05 | 0.05 | 0.05 | 0.05 |
| 83 - Hargraves Creek | 0.00 | 0.01 | 0.02 | 0.03 | 0.04 |
| 84 - Stanwell Creek | 0.01 | 0.01 | 0.02 | 0.03 | 0.03 |
| 85 - Flanagans Creek | 0.00 | 0.00 | 0.01 | 0.02 | 0.04 |
| 86 - Woodlands Creek | 0.00 | 0.01 | 0.01 | 0.02 | 0.03 |
| 87 - Slacky Creek | 0.00 | 0.01 | 0.01 | 0.03 | 0.04 |
| 88 - Bellambi Gully | 0.02 | 0.05 | 0.07 | 0.08 | 0.10 |
| 89 - Bellambi Lake | 0.03 | 0.06 | 0.10 | 0.12 | 0.14 |
| 90 - Towradgi Creek | 0.04 | 0.06 | 0.15 | 0.25 | 0.31 |
| 91 - Fairy Creek | 0.11 | 0.30 | 0.49 | 0.78 | 1.18 |
| 92 - Allans | 1.17 | 1.21 | 1.25 | 1.29 | 1.48 |
| 93 - PortKembla | 0.02 | 0.03 | 0.03 | 0.04 | 0.05 |
| 94 - Lake Illawarra | 35.83 | 35.97 | 36.63 | 38.54 | 41.56 |
| 95 - Elliott Lake | 0.08 | 0.10 | 0.13 | 0.27 | 0.68 |
| 96 - Minnamurra River | 1.86 | 2.06 | 2.85 | 3.87 | 4.84 |
| 97 - Spring Creek | 0.05 | 0.07 | 0.10 | 0.13 | 0.15 |
| 98 - Munna Munnora Creek | 0.00 | 0.03 | 0.05 | 0.06 | 0.07 |
| 99 - Werri Lagoon | 0.14 | 1.09 | 1.44 | 1.57 | 1.67 |
| 100 - Crooked River | 0.28 | 0.32 | 0.47 | 1.75 | 5.99 |
| 101 - Shoalhaven River | 31.90 | 42.46 | 71.38 | 100.50 | 114.44 |
| 102 - Wollumboola Lake | 6.33 | 8.71 | 9.17 | 9.64 | 10.01 |
| 103 - Currarong Creek | 0.03 | 0.07 | 0.11 | 0.21 | 0.57 |
| 104 - Cararma Creek | 2.39 | 2.49 | 3.06 | 4.02 | 4.37 |
| 105 - Wowly Gully | 0.16 | 0.53 | 1.13 | 1.29 | 1.43 |
| 106 - Callala Creek | 0.01 | 3.39 | 3.96 | 4.44 | 4.79 |
| 107 - Currambene Creek | 2.22 | 2.43 | 4.41 | 8.67 | 9.98 |
| 108 - Moona Creek | 0.14 | 3.05 | 4.26 | 4.66 | 4.95 |
| 109 - Flat Rock Creek | 0.01 | 0.08 | 0.26 | 0.37 | 0.46 |
| 110 - Captains Beach Lagoon | 0.05 | 0.14 | 0.17 | 0.19 | 0.21 |
| 111 - Telegraph Creek | 0.01 | 0.03 | 0.12 | 0.15 | 0.17 |
| 112 - JervisBay | 0.08 | 0.51 | 0.86 | 1.21 | 1.52 |
| 113 - St Georges Basin | 40.91 | 41.47 | 44.97 | 48.43 | 51.75 |
| 114 - Swan Lake | 4.68 | 5.98 | 6.35 | 6.71 | 6.96 |
| 115 - Berrara Creek | 0.26 | 0.87 | 0.97 | 1.04 | 1.08 |
| 116 - Nerrindillah Creek | 0.07 | 0.22 | 0.26 | 0.29 | 0.35 |
| 117 - Conjola Lake | 6.72 | 6.77 | 7.46 | 8.49 | 9.37 |
| 118 - Narrawallee Inlet | 1.04 | 1.35 | 3.38 | 5.02 | 6.12 |
| 119 - Mollymook Creek | 0.01 | 0.02 | 0.05 | 0.07 | 0.09 |
| 120 - Millards Creek | 0.00 | 0.03 | 0.04 | 0.05 | 0.06 |
| 121 - Ulladulla | 0.09 | 0.10 | 0.10 | 0.10 | 0.10 |
| 122 - Burrill Lake | 4.38 | 4.57 | 5.20 | 5.61 | 6.31 |
| 123 - Tabourie Lake | 1.49 | 1.49 | 1.55 | 2.00 | 2.66 |
| 124 - Termeil Lake | 0.57 | 1.28 | 1.48 | 1.61 | 1.70 |
| 125 - Meroo Lake | 1.37 | 2.11 | 2.58 | 2.77 | 2.89 |
| 126 - Willinga Lake | 0.31 | 1.22 | 1.69 | 1.89 | 2.01 |
| 127 - Butlers Creek | 0.03 | 0.07 | 0.14 | 0.17 | 0.21 |
| 128 - Durras Lake | 3.77 | 5.77 | 6.39 | 6.72 | 7.00 |
| 129 - Durras Creek | 0.02 | 0.59 | 0.76 | 0.84 | 0.90 |
| 130 - Maloneys Creek | 0.03 | 0.17 | 0.51 | 0.60 | 0.66 |
| 131 - Cullendulla Creek | 1.29 | 1.49 | 1.80 | 2.24 | 2.46 |
| 132 - Clyde River | 17.56 | 19.05 | 21.55 | 23.45 | 25.04 |
| 133 - BatemansBay | 0.06 | 0.54 | 0.80 | 2.00 | 2.95 |
| 134 - Saltwater Creek Rosedale | 0.00 | 0.01 | 0.01 | 0.05 | 0.09 |
| 135 - Tomaga River | 1.81 | 2.19 | 3.45 | 4.88 | 5.45 |
| 136 - Candlagan Creek | 0.20 | 0.23 | 0.56 | 2.28 | 2.82 |
| 137 - Bengello Creek | 0.01 | 0.02 | 0.06 | 0.09 | 0.12 |
| 138 - Moruya River | 6.14 | 7.41 | 10.06 | 13.00 | 15.24 |
| 139 - Congo Creek | 0.13 | 3.40 | 4.40 | 4.83 | 5.20 |
| 140 - Meringo Creek | 0.08 | 0.12 | 0.20 | 0.25 | 0.28 |
| 141 - Kellys Lake | 0.06 | 0.06 | 0.16 | 0.21 | 0.24 |
| 142 - Coila Lake | 7.12 | 8.21 | 8.45 | 8.73 | 8.98 |
| 143 - Tuross River | 15.50 | 16.48 | 19.07 | 22.21 | 25.55 |
| 144 - Lake Brunderee | 0.21 | 0.99 | 1.15 | 1.21 | 1.25 |
| 145 - Lake Tarourga | 0.33 | 0.43 | 0.49 | 0.52 | 0.56 |
| 146 - Lake Brou | 2.45 | 3.86 | 4.06 | 4.21 | 4.44 |
| 147 - Lake Mummuga | 1.65 | 2.25 | 2.35 | 2.47 | 2.54 |
| 148 - Kianga Lake | 0.17 | 0.18 | 0.28 | 0.31 | 0.34 |
| 149 - Wagonga Inlet | 6.94 | 7.16 | 7.55 | 8.08 | 8.56 |
| 150 - Little Lake Narooma | 0.10 | 0.14 | 0.17 | 0.21 | 0.23 |
| 151 - Bullengella Lake | 0.15 | 0.16 | 0.18 | 0.19 | 0.21 |
| 152 - Nangudga Lake | 0.74 | 1.22 | 1.30 | 1.36 | 1.43 |
| 153 - Corunna Lake | 2.13 | 2.69 | 2.87 | 2.98 | 3.09 |
| 154 - Tilba Lake | 1.17 | 1.59 | 1.80 | 1.90 | 1.97 |
| 155 - Little Lake Wallaga | 0.13 | 0.17 | 0.19 | 0.21 | 0.23 |
| 156 - Wallaga Lake | 9.31 | 9.33 | 10.03 | 11.52 | 12.59 |
| 157 - Bermagui River | 2.16 | 2.49 | 2.87 | 3.52 | 3.86 |
| 158 - Baragoot Lake | 0.55 | 0.84 | 0.92 | 0.98 | 1.04 |
| 159 - Cuttagee Lake | 1.35 | 2.04 | 2.15 | 2.23 | 2.30 |
| 160 - Murrah River | 0.84 | 0.98 | 1.11 | 1.48 | 2.03 |
| 161 - Bunga Lagoon | 0.14 | 0.20 | 0.24 | 0.26 | 0.34 |
| 162 - Wapengo Lagoon | 3.67 | 3.87 | 4.13 | 4.67 | 5.02 |
| 163 - Middle Lagoon | 0.56 | 1.08 | 1.20 | 1.29 | 1.39 |
| 164 - Nelson Lagoon | 1.35 | 1.48 | 1.61 | 1.91 | 1.99 |
| 165 - Bega River | 3.84 | 4.36 | 4.74 | 5.25 | 5.77 |
| 166 - Wallagoot Lake | 3.98 | 5.14 | 5.40 | 5.63 | 5.78 |
| 167 - Bournda Lagoon | 0.08 | 0.19 | 0.20 | 0.21 | 0.22 |
| 168 - Back Lagoon | 0.38 | 0.74 | 0.81 | 0.84 | 0.88 |
| 169 - Merimbula Lake | 5.58 | 5.73 | 6.03 | 6.68 | 7.17 |
| 170 - Pambula River | 4.72 | 4.94 | 5.47 | 6.43 | 7.07 |
| 171 - Curalo Lagoon | 0.80 | 0.83 | 0.91 | 1.18 | 1.32 |
| 172 - Shadrachs Creek | 0.01 | 0.11 | 0.12 | 0.13 | 0.14 |
| 173 - Nullica River | 0.34 | 2.83 | 3.08 | 3.16 | 3.22 |
| 174 - Boydtown Creek | 0.02 | 0.20 | 0.23 | 0.26 | 0.33 |
| 175 - Towamba River | 2.04 | 2.34 | 2.68 | 3.12 | 3.59 |
| 176 - Fisheries Creek | 0.09 | 0.35 | 0.36 | 0.37 | 0.38 |
| 178 - Saltwater Creek Eden | 0.06 | 0.21 | 0.23 | 0.24 | 0.26 |
| 179 - Woodburn Creek | 0.05 | 0.31 | 0.33 | 0.36 | 0.38 |
| 180 - Wonboyn River | 4.21 | 4.38 | 4.83 | 5.88 | 6.16 |
| 181 - Merrica River | 0.12 | 0.18 | 0.19 | 0.19 | 0.20 |
| 182 - Table Creek | 0.06 | 0.21 | 0.23 | 0.25 | 0.27 |
| 183 - Nadgee River | 0.28 | 1.57 | 1.65 | 1.72 | 1.79 |
| 184 - Nadgee Lake | 1.20 | 1.43 | 1.47 | 1.54 | 1.62 |
| Grand Total | 1607 | 2019 | 2788 | 3442 | 3922 |
